# Antimicrobial Resistance Trends, Resistance Mechanisms, and Antibiotic Consumption in COVID-19 Versus Non-COVID-19 Units: A Seven-Year Retrospective Cohort Study

**DOI:** 10.3390/antibiotics14111149

**Published:** 2025-11-13

**Authors:** Stefan Porubcin, Alena Rovnakova, Ondrej Zahornacky, Pavol Jarcuska

**Affiliations:** 1 The Department of Infectious Diseases and Travel Medicine, Louis Pasteur University Hospital, Rastislavova 43, 04011 Kosice, Slovakia; stefan.porubcin@unlp.sk (S.P.); ondrej.zahornacky@unlp.sk (O.Z.); pavol.jarcuska@upjs.sk (P.J.); 2 Faculty of Medicine, Pavol Jozef Safarik University in Kosice, Tr. SNP No 1, 04001 Kosice, Slovakia

**Keywords:** COVID-19, antimicrobial resistance, antibiotic consumption, *Klebsiella*, *Acinetobacter*, *P. aeruginosa*, carbapenemase, stewardship

## Abstract

**Background:** The COVID-19 pandemic profoundly affected healthcare delivery and antibiotic prescribing, raising concerns about increasing antimicrobial resistance. This study investigated seven-year trends in bacterial resistance, underlying resistance mechanisms, and antibiotic consumption in COVID-19 and non-COVID-19 units at a tertiary hospital in Slovakia. **Methods**: A retrospective cohort analysis (2018–2024) was conducted using clinical isolates of *Klebsiella* sp., *Acinetobacter* sp., and *P. aeruginosa*. Data on hospitalizations, resistance profiles, resistance mechanisms, and standardized antibiotic use were compared between COVID-19 and non-COVID-19 departments. **Results**: Hospitalizations markedly decreased in COVID-19 units, while pathogen occurrence—particularly of *Acinetobacter* sp.—was substantially higher compared with non-COVID-19 units. Resistance in *Klebsiella* sp. shifted from extended-spectrum beta-lactamase production to carbapenemase production. *Acinetobacter* sp. remained highly resistant, although some declines were observed in ceftazidime and gentamicin resistance. *P. aeruginosa* showed a gradual reduction in resistance, notably to piperacillin/tazobactam and imipenem. Antibiotic consumption was consistently higher in COVID-19 units, particularly for broad-spectrum beta-lactams and carbapenems, whereas fluoroquinolone use decreased over time. Clinically effective treatment options were considerably fewer in COVID-19 units, often limited to colistin. **Conclusions**: COVID-19 units experienced greater pathogen burden, higher broad-spectrum antibiotic exposure, and increased prevalence of critical resistance mechanisms. Tailored antimicrobial stewardship and infection prevention, and control are essential to reduce selective pressure and preserve last-line antibiotics.

## 1. Introduction

On 11 March 2020, the World Health Organization (WHO) declared coronavirus disease 2019 (COVID-19) a global pandemic, resulting in substantial worldwide mortality. On 5 May 2023, WHO announced that COVID-19 no longer constituted a public health emergency of international concern. Parallel to the COVID-19 pandemic, Europe has faced a “silent” but equally serious pandemic of antimicrobial resistance (AMR), largely driven by excessive and inappropriate antibiotic use. Most antibiotics in Europe are consumed in the community, particularly in primary care. The convergence of COVID-19 and AMR intensified existing weaknesses in infection control and antimicrobial stewardship. Despite the viral etiology of COVID-19, antibiotic prescribing increased in the United States, often motivated by concerns about bacterial coinfections. By contrast, data from the European Union (EU) suggest the opposite trend, with outpatient antibiotic prescribing declining in 2019–2020. Factors such as altered transmission dynamics, changes in healthcare-seeking behavior, modified prescribing practices, and reduced incidence of non-COVID-19 respiratory infections likely contributed. In hospitals, however, overall antibiotic consumption decreased in 2020–2021, while the use of broad-spectrum and last-line agents markedly increased. More than 60% of COVID-19 patients with confirmed bacterial infections harbored resistant isolates [1,2].

The Centers for Disease Control and Prevention (CDC) analyzed the pandemic’s impact on AMR in the United States. Between 2012 and 2017, AMR-related deaths declined by 18%. This trend reversed with the pandemic: in 2020, nosocomial AMR infections and associated deaths rose by 15% compared with 2019. Over 29,400 individuals died from AMR infections in the first pandemic year, nearly 40% acquired during hospitalization. Approximately 80% of hospitalized COVID-19 patients received antibiotics between March and October 2020, despite the absence of bacterial infection. The pandemic disrupted AMR surveillance, with CDC lacking data for 9 of 18 priority pathogens, likely underestimating the true burden. Overcrowded hospitals, prolonged hospitalizations, and increased invasive procedures (e.g., ventilation, catheterization) elevated infection risk. Shortages of protective equipment, reduced staffing, and supply chain disruptions hindered infection prevention. Prioritization of COVID-19 testing and treatment delayed diagnosis of multi-drug resistant (MDR) infections. Notably, carbapenem-resistant *Acinetobacter baumannii* (CR-AB) infections increased by 78% in 2020 compared with 2019, *Candida auris* by 60%, and other *Candida* species by 26%. The pandemic compromised CDC strategies in three domains:

- Surveillance—23% fewer samples processed in 2020 than 2019;
- Infection prevention—disrupted routines facilitated resistant pathogen spread;
- Antibiotic use—increased prescribing despite limited indications [3].

After 2020, the pandemic profoundly affected outpatient prescribing, infection control, and resource allocation, influencing AMR trends. Several studies suggest accelerated dissemination of extended-spectrum β-lactamase (ESBL)-producing *Enterobacterales* and carbapenem-resistant *Enterobacterales* (CRE) [4]. Approximately 75% of COVID-19 patients received antibiotics, though <10% had confirmed bacterial co-infections [5]. Such overuse exacerbates AMR. The absence of antimicrobial stewardship programs (ASPs) was linked to increased resistance in Gram-negative bacteria. CDC reported a 15% rise in resistant infections, including CR-AB, methicillin-resistant *Staphylococcus aureus* (MRSA), and CRE, underscoring the need for improved monitoring and prevention [6].

Trends in Gram-positive resistance remained relatively stable. Meta-analyses showed no significant change in MRSA or vancomycin-resistant *Enterococcus* (VRE). In contrast, Gram-negative AMR rose sharply. The pooled incidence of resistant Gram-negative organisms –including ESBL-producing *Enterobacterales*, CRE, carbapenem-resistant *P. aeruginosa* (CR-PA), and CR-AB—was 1.64 (95% CI: 0.92–2.92), with a relative risk (RR) of 1.08 (95% CI: 0.91–1.29). Lack of ASPs was associated with further increases, with RR 1.11 (95% CI: 1.03–1.20), highlighting antibiotic selective pressure and nosocomial transmission [4].

Several factors shaped AMR trends. Initial pandemic measures—hand hygiene, distancing, restricted facility access—may have temporarily reduced AMR, especially in 2020. However, disruption of infection control and ASPs likely offset these gains. Overuse of broad-spectrum antibiotics, particularly β-lactams, created selective pressure favoring resistant Gram-negative organisms [7,8].

Our study investigated the impact of the COVID-19 pandemic and ward repurposing on hospitalization dynamics, antibiotic consumption, and resistance trends of *Klebsiella* sp., *Acinetobacter* sp., and *P. aeruginosa* at the Louis Pasteur University Hospital in Košice between 2018 and 2024. By comparing COVID-19 and non-COVID-19 units across pre-pandemic, pandemic, and post-pandemic periods, we aimed to assess how healthcare restructuring and shifts in antibiotic use influenced resistance epidemiology in a tertiary care setting.

## 2. Results

### 2.1. Hospitalization Trends

Hospitalization dynamics from 2018 to 2024 exhibited distinct patterns, reflecting the influence of the COVID-19 pandemic and the repurposing of bed capacity.

In the COVID-19 units (4th DIM and 2nd DS), hospitalization numbers remained stable during the pre-pandemic period (2018–2019). However, beginning in 2020, a notable decline occurred, reaching the lowest point in 2021 (approximately 1200 hospitalizations). This decrease was statistically significant according to both linear regression and correlation analyses (Table 1). In the following years, hospitalization numbers gradually returned to pre-pandemic levels.

**Figure 1:**
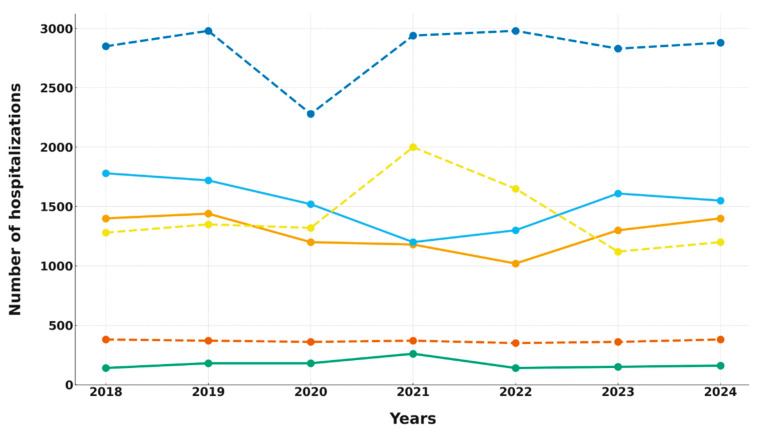
Hospitalization trends in COVID-19 and non-COVID-19 units. Solid lines denote COVID-19 units; dashed lines denote non-COVID-19 units. Color key: blue—4th DIM (solid), orange—2nd DS (solid), green—CAIC (solid), red—1st DIM (dashed), purple—1st DS (dashed), brown—DAIC (dashed).

In CAIC, a temporary surge in hospitalizations was observed in 2021 during the Delta wave, with an increase of about 40% compared to previous years. This was followed by stabilization at pre-pandemic levels (around 140–150 hospitalizations annually), with no significant long-term trend detected (Table 1 and Figure 1).

The non-COVID-19 units displayed a different pattern. The 1st DIM experienced a temporary rise in hospitalizations in 2021 due to patient transfers from repurposed wards. The 1st DS showed a brief decline in 2020, followed by a recovery to approximately 2900 hospitalizations annually. DAIC maintained stable hospitalization numbers (342–379 annually), indicating minimal disruption related to the pandemic. Statistical analyses did not reveal significant long-term trends in these units (Table 1).

In summary, only the COVID-19 units (4th DIM and 2nd DS) demonstrated a statistically significant decline in hospitalizations, while CAIC and the non-COVID-19 departments (1st DIM, 1st DS, DAIC) experienced only temporary, non-significant fluctuations. These findings highlight the heterogeneous effects of the pandemic and ward repurposing on hospitalization patterns.

### 2.2. Comparison of Pathogen Occurrence Across Departments and Trends over Time

The analysis of pathogen occurrence between 2018 and 2024 revealed clear contrasts between COVID-19 and non-COVID-19 units, although most interdepartmental differences did not reach statistical significance.

When comparing paired departments (4th DIM vs. 1st DIM; 2nd DS vs. 1st DS; CAIC vs. DAIC), no significant differences in pathogen occurrence were confirmed by the Wilcoxon test (all *p* = 0.25), likely due to the limited number of evaluated periods and high variability of data, particularly in the CAIC.

Nevertheless, numerical disparities were apparent. Among surgical departments, the 2nd DS consistently showed higher occurrence of all evaluated pathogens compared with the 1st DS (e.g., *P. aeruginosa* 10.7–6.8 vs. 2.1–3.3 isolates per 100 patients). Similarly, in intensive care departments, pathogen occurrence was markedly higher in the CAIC (*Acinetobacter* sp. 133.9–28.1/100 patients) than in the DAIC (17.6–10.4/100 patients), although without statistical significance.

Significant results were observed mainly in the time-series analysis. Decreasing trends were identified for *Acinetobacter* sp. in the 4th DIM and CAIC, for *Klebsiella* sp. in the 1st and 4th DIM, and for *P. aeruginosa* in the 2nd DS (Table 2).

In summary, despite evident numerical differences—particularly between the CAIC and DAIC, and between the 2nd DS and 1st DS—statistical testing did not confirm significant interdepartmental differences in pathogen occurrence. By contrast, time-series analysis revealed several significant decreasing trends, mainly in internal medicine and surgical COVID-19 units as well as in the CAIC.

### 2.3. Comparison of Pathogen Occurrence During the Pandemic (2020–2022) Between COVID-19 and Non-COVID-19 Units

The analysis of ward repurposing during the pandemic period (2020–2022) demonstrated that the occurrence of nosocomial pathogens was consistently and significantly higher in COVID-19 units compared with non-COVID-19 units. The most pronounced differences were observed for *Acinetobacter* sp., which showed a markedly higher proportion of positive clinical samples in COVID-19 units across all evaluated years. The same pattern was evident for *Klebsiella* sp., where the proportion of positive isolates was consistently almost twice as high in COVID-19 units compared with non-COVID-19 units. For *P. aeruginosa*, differences were less pronounced but remained consistent throughout the pandemic period, with higher rates in COVID-19 units across all three years analyzed.

Overall, the results confirmed a significantly greater occurrence of all three pathogens in COVID-19 units during 2020–2022 (Table 3).

### 2.4. Resistance Trends of Klebsiella sp. in COVID-19 vs. Non-COVID-19 Units

The analysis of *Klebsiella* sp. resistance across internal medicine, surgical, and intensive care units between 2018 and 2024 revealed marked differences between COVID-19 and non-COVID-19 units, along with dynamic changes during the pandemic (Table 4).

In the 4th DIM, resistance to cephalosporins and fluoroquinolones showed a declining trend, while resistance to β-lactam/β-lactamase inhibitor combinations and carbapenems increased during the pandemic and remained elevated thereafter. The 1st DIM followed a similar pattern, with decreasing resistance to gentamicin and ciprofloxacin but rising resistance to ertapenem, which appeared during the pandemic and persisted in the post-pandemic years.

In the 2nd DS, the pandemic period was associated with a sharp increase in resistance across multiple antibiotic classes, particularly cephalosporins, β-lactam/β-lactamase inhibitor combinations, and carbapenems. Post-pandemic data indicated partial declines; however, resistance to ertapenem and cefoperazone/sulbactam combinations remained significantly higher than pre-pandemic levels. The 1st DS exhibited temporary reductions in cephalosporin and gentamicin resistance during the pandemic, followed by a rebound, thereafter, accompanied by a transient rise in amikacin resistance.

The CAIC displayed the highest resistance rates overall, with pre-pandemic values for cephalosporins and ciprofloxacin exceeding 70%. Although partial post-pandemic declines were observed, resistance remained high. A notable and consistent decrease was recorded for gentamicin. In contrast, the DAIC maintained lower baseline resistance levels but demonstrated increasing trends for piperacillin/tazobactam, cefoperazone/sulbactam, and carbapenems, while gentamicin resistance declined.

Overall, *Klebsiella* sp. resistance to cephalosporins and fluoroquinolones was higher in COVID-19 units, whereas the pandemic period was characterized by a substantial rise in resistance to β-lactam/β-lactamase inhibitor combinations and carbapenems. These increases were only partially reversed post-pandemically, while gentamicin resistance declined consistently across all unit types (Table 4).

### 2.5. Resistance Trends of Acinetobacter sp. in COVID-19 vs. Non-COVID-19 Units

The analysis of *Acinetobacter* sp. resistance across internal medicine, surgical, and intensive care units between 2018 and 2024 demonstrated persistently high resistance rates across most departments, with clear differences between COVID-19 and non-COVID-19 units (Table 5).

In the 4th DIM, resistance remained consistently high for most antibiotics, including piperacillin/tazobactam and ceftazidime, with no statistically significant trends identified. In contrast, the 1st DIM exhibited a significant increase in ampicillin/sulbactam resistance during and after the pandemic.

Among surgical units, the 2nd DS showed extremely high resistance levels across nearly all antibiotics, accompanied by a significant rise in ampicillin/sulbactam resistance during the pandemic period. The 1st DS displayed a variable pattern, with resistance decreasing before the pandemic, then rising and stabilizing at high levels thereafter. A statistically significant increase in ampicillin/sulbactam resistance was confirmed in this unit as well.

The highest resistance levels were observed in the intensive care units. The CAIC exhibited exceptionally high resistance across all antibiotics, often exceeding 90%, but significant post-pandemic decreases were observed for ceftazidime and gentamicin, while ciprofloxacin resistance showed a borderline decline. In contrast, the DAIC maintained high but relatively stable resistance rates, with significant upward trends in ampicillin/sulbactam, gentamicin, and amikacin resistance.

In summary, *Acinetobacter* sp. demonstrated persistently high resistance across all departments, with partial post-pandemic improvements in the CAIC, but increasing ampicillin/sulbactam resistance across multiple units. Statistically significant trends included decreasing ceftazidime and gentamicin resistance in the CAIC, contrasted by rising ampicillin/sulbactam resistance in several other departments (Table 5).

### 2.6. Resistance Trends of P. aeruginosa in COVID-19 vs. Non-COVID-19 Units

Across internal medicine, surgical, and intensive care units between 2018 and 2024, *P. aeruginosa* resistance generally displayed a declining trend, particularly for β-lactams and ciprofloxacin, although the magnitude varied by department type (Table 6).

In the 4th DIM, resistance remained largely stable without statistically significant changes, despite numerical decreases across most antibiotic classes. In the 1st DIM, significant reductions were observed for ciprofloxacin, cefepime, and gentamicin, indicating a gradual improvement in susceptibility during and after the pandemic.

Among surgical units, the 2nd DS exhibited significant decreases in resistance to multiple β-lactams, including cefepime, ceftazidime, and piperacillin/tazobactam, as well as to gentamicin and ciprofloxacin. In contrast, the 1st DS showed only non-significant numerical reductions across most antibiotics.

The most pronounced changes were recorded in intensive care. In the CAIC, marked and statistically significant declines were documented for piperacillin/tazobactam, cefepime, gentamicin, and ciprofloxacin, indicating a broad reduction in multidrug resistance. Similarly, the DAIC (non-COVID-19 unit) showed significant downward trends for cefepime, imipenem, gentamicin, and ciprofloxacin, confirming improved antimicrobial susceptibility patterns over time.

Across all departments, ciprofloxacin remained the most problematic agent, with resistance frequently exceeding 35–45%, although significant decreases were confirmed in several units. In contrast, amikacin consistently maintained the lowest resistance, while colistin resistance was virtually absent throughout the entire study period.

Overall, *P. aeruginosa* resistance across the monitored departments demonstrated a consistent downward trend between 2018 and 2024, particularly for cephalosporins, carbapenems, and aminoglycosides. The most significant reductions were recorded in COVID-19 units (CAIC, 2nd DS) and, to a lesser extent, in non-COVID-19 units (1st DIM, DAIC). These findings indicate progressive improvements in the epidemiological situation, with intensive care units showing the most dynamic and statistically supported declines (Table 6).

### 2.7. Trends in Pathogen-Specific Resistance Mechanisms

The evaluation of resistance mechanisms demonstrated distinct patterns between COVID-19 and non-COVID-19 units, characterized by several significant, pathogen-specific alterations observed throughout the pandemic period.

For *Klebsiella* sp., ESBL prevalence declined significantly in both settings, more markedly in COVID-19 units, while carbapenemase/MBL production increased in parallel, indicating a shift from ESBL towards more advanced resistance mechanisms. Inter-unit comparison confirmed significant differences: narrow-spectrum β-lactamase production was more prevalent in non-COVID-19 units, whereas carbapenemase/MBL production, aminoglycoside resistance, and multidrug resistance occurred more frequently in COVID-19 wards.

For *Acinetobacter* sp., there were no significant changes over time in the rates of carbapenem or multidrug resistance between 2018 and 2022. Nevertheless, both resistance mechanisms remained significantly more common in COVID-19 units, underscoring a more severe and persistent epidemiological challenge in these wards.

For *P. aeruginosa*, COVID-19 units exhibited significant downward trends across multiple mechanisms, including efflux-porin alterations, carbapenemase/MBL production, carbapenem resistance, and multidrug resistance. Non-COVID-19 units also showed significant reductions in carbapenem and multidrug resistance. Despite these improvements, the overall prevalence of all monitored mechanisms remained higher in COVID-19 units.

In summary, the pandemic period was associated with distinct pathogen-specific shifts: *Klebsiella* sp. transitioned from ESBL to carbapenemase dominance, *P. aeruginosa* demonstrated broad reductions across resistance mechanisms, and *Acinetobacter* sp. maintained persistently high resistance levels with consistently greater prevalence in COVID-19 units (Table 7).

### 2.8. Antibiotic Consumption Trends Between COVID-19 and Non-COVID-19 Units

The analysis of standardized antibiotic consumption per 100 hospitalizations revealed substantial differences between COVID-19 and non-COVID-19 units, with overall use markedly higher in COVID-19 wards.

During the pandemic period, the most pronounced increases were recorded for broad-spectrum β-lactam/β-lactamase inhibitor combinations, particularly piperacillin/tazobactam and cefoperazone/sulbactam, both showing sharp surges in 2020–2021, especially in COVID-19 units.

In contrast, aminoglycoside consumption demonstrated a steady decline in both settings, most notably for amikacin, while gentamicin remained relatively stable but trended downward in COVID-19 units after 2020.

Among carbapenems, meropenem displayed the most striking pattern, with a dramatic surge during the pandemic in COVID-19 units, followed by a gradual post-pandemic decline. Imipenem and ertapenem both showed decreasing utilization over time, more pronounced in COVID-19 wards.

Within cephalosporins, ceftriaxone consumption remained consistently higher in COVID-19 units throughout the study period, exceeding levels observed in non-COVID-19 units more than twofold. Conversely, ciprofloxacin use decreased sharply across all units, reflecting a sustained decline in fluoroquinolone prescribing.

The most notable post-pandemic increase was observed for colistin, whose consumption rose substantially in 2022, particularly in COVID-19 units, indicating more frequent use as a last-line option against multidrug-resistant pathogens.

**Figure 2:**
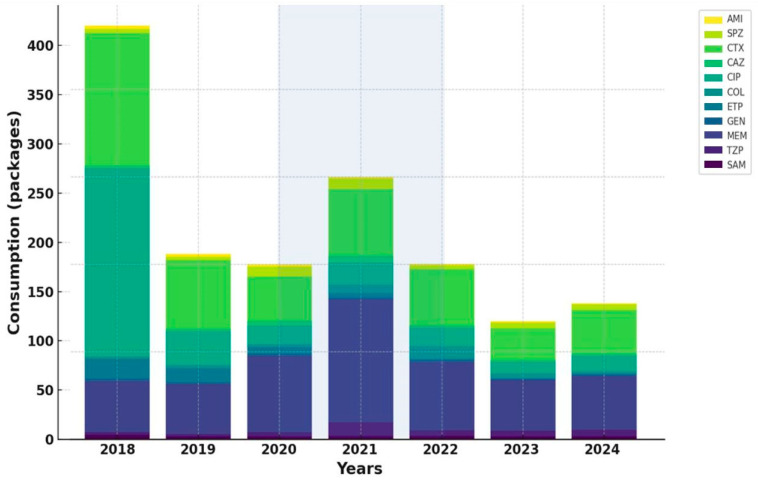
Trends in the adjusted antibiotic consumption in COVID-19 units.

**Figure 3:**
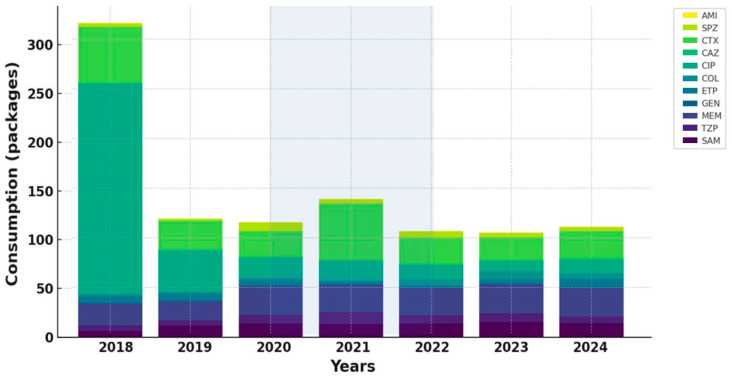
Trends in the adjusted antibiotic consumption in non-COVID-19 units.

Overall, antibiotic consumption patterns mirrored the epidemiological dynamics observed during the pandemic, characterized by intensified use of broad-spectrum β-lactams and carbapenems in COVID-19 units, followed by gradual post-pandemic normalization and selective increase in last-resort agents (Figure 2 and Figure 3).

### 2.9. Correlation Between Antibiotic Consumption and Resistance Mechanisms

The analysis of correlations between antibiotic consumption and the prevalence of major resistance mechanisms in *Klebsiella* sp. demonstrated that changes in antibiotic use were directly associated with shifts in resistance, particularly during the pandemic period.

In both COVID-19 and non-COVID-19 units, declining use of ceftriaxone and ciprofloxacin was accompanied by a marked reduction in ESBL prevalence. A significant positive correlation was confirmed between ciprofloxacin consumption and ESBL occurrence, whereas no association was observed for ceftriaxone. In contrast, increased meropenem consumption coincided with a rise in carbapenemase-mediated resistance, showing a borderline-significant trend in COVID-19 units and a non-significant association in non-COVID-19 units.

For *Acinetobacter* sp., correlations were not assessed due to the absence of significant resistance mechanism trends, and no significant associations were identified between antibiotic consumption and resistance mechanisms in *P. aeruginosa*.

Overall, the most pronounced and statistically supported association was observed between declining ciprofloxacin consumption and the reduction of ESBL resistance in *Klebsiella* sp. across both COVID-19 and non-COVID-19 units. Additionally, COVID-19 wards demonstrated a near-significant trend linking increased meropenem consumption with rising carbapenemase resistance (Table 8).

### 2.10. Comparison of Clinical Applicability of Antibiotics Between COVID-19 and Non-COVID-19 Units

Non-COVID-19 departments retained a broader range of effective antibiotics against *Klebsiella* sp. and *P. aeruginosa*, whereas therapeutic options in COVID-19 units—particularly in the CAIC and 2nd DS—were markedly restricted.

For *Klebsiella* sp., non-COVID-19 units (1st DIM, 1st DS, DAIC) preserved up to seven effective antibiotics, while COVID-19 units CAIC and 2nd DS were limited to colistin as the only viable option. The 4th DIM maintained six active agents.

For *Acinetobacter* sp., the situation was critical across all units, with colistin being the sole effective antibiotic, highlighting extensive multidrug resistance.

**Figure 4:**
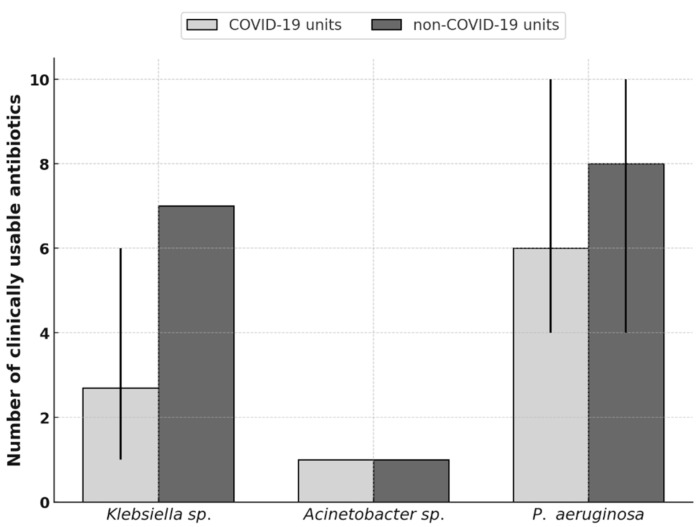
Comparison of clinically usable antibiotics between COVID-19 and non-COVID-19 units. Bars show the mean number of clinically applicable antibiotics (≤20% resistance) for each pathogen; thin vertical lines indicate the departmental range.

For *P. aeruginosa*, differences were most pronounced: non-COVID-19 units (1st DIM, 1st DS) retained up to ten antibiotics with resistance below 20%, whereas DAIC and COVID-19 units (CAIC, 2nd DS) were limited to four. The 4th DIM was an exception among COVID-19 wards, maintaining ten usable antibiotics—comparable to non-COVID-19 units (Figure 4).

Among individual agents, ceftriaxone, ciprofloxacin, and gentamicin were clinically unusable in most settings, with resistance exceeding 20%. Piperacillin/tazobactam, cefoperazone/sulbactam, and meropenem remained effective mainly in non-COVID-19 units but had already lost reliability in CAIC and 2nd DS. Amikacin preserved activity against *Klebsiella* sp. and partly against *P. aeruginosa* in non-COVID-19 units, though it was ineffective against *Acinetobacter* sp. Ciprofloxacin showed resistance consistently above 20% across all pathogens and units, rendering it clinically obsolete.

Overall, COVID-19 units were disproportionately burdened with multidrug-resistant strains, leaving only limited therapeutic options, while non-COVID-19 departments preserved broader antibiotic efficacy and greater flexibility in empirical and targeted therapy.

## 3. Discussion

The pandemic unevenly affected hospital operations across COVID-19 and non-COVID-19 units (2018–2024). COVID-19 wards (4th DIM, 2nd DS) maintained stable admissions in 2018–2019, followed by a ≈30% decline in 2020–2021, consistent with international data showing a 20–50% drop in elective care and with nationwide restrictions on non-urgent procedures, such as those implemented in Canada from March 2020 [9,10,11,12]. Hospitalisations gradually recovered from 2022, approaching pre-pandemic levels by 2023–2024, though Canadian studies still noted a 10–15% deficit [12]. In contrast, the CAIC reported a >40% surge in 2021, reflecting the global rise in intensive care demand, comparable to pandemic epicentres where ICU capacity expanded by >50%, and up to 80% of ICU beds in Italy were occupied by COVID-19 patients during the first wave [13,14].

Non-COVID-19 units were mainly influenced by patient-flow redistribution and compensatory transfers. The 1st DIM showed a 50% rise in 2021, consistent with the hub-and-spoke reallocation model [11,14]. The 1st DS rebounded from a transient 2020 decline, stabilising near 2900 annual admissions. The DAIC remained stable, indicating minimal disruption and a supporting role similar to other ICUs [15]. Overall, COVID-19 units experienced marked but transient declines, whereas non-COVID-19 units were relatively stable. The convergence of long-term volumes between 4th and 1st DIM suggests these disparities were limited to the pandemic period, with remaining differences among surgical and intensive care units likely reflecting patient acuity, referral patterns, and capacity rather than the pandemic itself.

Comparative analysis of *Klebsiella* sp., *Acinetobacter* sp., and *P. aeruginosa* across departments revealed numerical differences, with higher isolation rates in the 2nd DS versus 1st DS, and greater values in the CAIC than in the DAIC, although none reached statistical significance. These findings correspond with the recognised epidemiological burden of surgical and intensive care settings, where mechanical ventilation, prolonged hospitalisation, and extensive broad-spectrum antibiotic exposure promote persistence and spread of multidrug-resistant organisms (MDROs) [16,17,18].

Time-series analysis identified statistically significant declines in pathogen occurrence: *Acinetobacter* sp. in the 4th DIM and CAIC, *Klebsiella* sp. in both the 1st DIM and 4th DIM, and *P. aeruginosa* in the 2nd DS. These trends mirror international data showing decreasing MDRO incidence over the past decade, attributed to reinforced infection prevention, antimicrobial stewardship, and greater awareness [17,19]. Despite this decline, MDROs remain a critical challenge in high-acuity environments, particularly in intensive care units.

During the pandemic, COVID-19 units exhibited a markedly higher MDRO burden, most notably for *Acinetobacter* sp., which showed a more than sevenfold increase compared with non-COVID-19 units. *Klebsiella* sp. and *P. aeruginosa* also displayed two- to threefold higher rates across 2020–2022. These findings are consistent with global reports of increased MDRO prevalence in COVID-19 ICUs, where *Acinetobacter* sp. predominated. Likely contributors included excessive patient load, frequent invasive ventilation, disruption of infection-prevention measures due to limited resources, and prolonged empirical broad-spectrum therapy for presumed bacterial co-infection [17].

Before the pandemic, *Klebsiella* sp. across departments displayed the expected multifactorial resistance profile. Internal medicine wards reported 40–50% resistance to third-generation cephalosporins (ceftriaxone, ceftazidime), consistent with ≈50% ESBL prevalence, with the 4th DIM trending higher but without significance. Surgical units differed, with 55% resistance in the 2nd DS versus 36% in the 1st DS, reflecting other hospital prevalences often >50% [20]. During the pandemic, cephalosporin resistance on internal medicine wards fluctuated without significance: resistance in the 2nd DS remained high, whereas the 1st DS declined toward 20%. Literature reports both increases and up to 10% ESBL reductions in specific wards, underscoring local effects of policy and decreased elective activity [21,22]. This pattern likely reflects reduced third-generation cephalosporin use, consistent with known associations between lower consumption and attenuated resistance [23].

According to 2023 ECDC–WHO surveillance, *Klebsiella* sp. remains a key resistant pathogen in Europe. Invasive infections have increased since 2019, and carbapenem resistance now exceeds the EU 2030 reduction target. About half of the countries report rates > 50%, highest in southern and eastern Europe, while northern regions show relative stabilisation. Overall, EU/EEA trends indicate continued transmission of carbapenem-resistant *Klebsiella* strains [6].

Intensive care reflected surgical epidemiology: the repurposed CAIC reached ≈70% cephalosporin resistance versus 30% in the DAIC, likely driven by a pre-existing AMR burden and mixed surgical case profiles. Frequent transfers from the repurposed 2nd DS (55% resistance) plausibly maintained transmission, as shown in other studies [24]. Despite reduced cephalosporin use, persistence via horizontal gene transfer and environmental reservoirs remains likely [25]. Conversely, the non-repurposed DAIC, epidemiologically linked to the 1st DS (36% resistance), maintained lower rates, supported by steadier stewardship and a more homogeneous patient population [25].

Carbapenem resistance remains a critical global issue [26]. During the pandemic, ertapenem resistance rose sharply in the 2nd DS (meropenem stable), with smaller increases in the 1st DS, 4th DIM, and 1st DIM. The CAIC recorded the highest rates, while the DAIC remained lower but increased slightly. Similar surges were reported in Romania, Germany, and China, linked to intensified carbapenem use in COVID-19 wards [20,22,27,28]. Greater antibiotic pressure in repurposed units likely drove these patterns, as seen with meropenem spikes in Turkey and rising ertapenem resistance in Ireland [29,30].

Increasing resistance to β-lactam/β-lactamase inhibitors further limits empiric options [31]. Piperacillin/tazobactam resistance increased in the 4th DIM, 2nd DS, 1st DIM, and DAIC, indicating that COVID-19 accelerated antimicrobial resistance and that ventilated ICU patients show high rates [20,32]. In contrast, Iranian data reported strong post-pandemic piperacillin/tazobactam activity against multidrug-resistant *Klebsiella* sp. [33]. Locally, resistance stabilised in internal medicine wards, decreased in the CAIC, and rose in the DAIC. Cefoperazone/sulbactam resistance also increased, notably in the 2nd DS and DAIC, though Taiwanese studies confirm its activity against ESBL-producing *Klebsiella* sp. [34]. These increases may encourage carbapenem overuse, reinforcing resistance; antibiotic rotation may mitigate this [35].

Aminoglycosides remain essential for severe *Klebsiella* sp. infections [36]. Globally, resistance rose to ≈23% during the pandemic [37]. In UNLP, gentamicin resistance averaged ≈40% on repurposed wards (up to ≈70% in the CAIC) versus ≈30% elsewhere, while amikacin resistance stayed low (≈3%). Gentamicin resistance declined significantly in the 4th DIM, CAIC, 1st DIM, and DAIC, opposite to trends in Jordan and Argentina, likely reflecting reduced use and nephrotoxicity concerns [38,39,40]. Persistently low amikacin resistance reflects limited use and fewer enzyme-mediated changes [41]. Pre-pandemic, resistance was higher in the 2nd DS (14.8%) and CAIC (45.6%), consistent with U.S. data, while non-repurposed units such as the DAIC stayed <10%, similar to Jordanian estimates (11%) [42,43]. Only the 1st DS showed a significant post-pandemic rise, possibly due to mobile *aac(6′)-Ib* and co-selection with *blaKPC* and *qnrB19* [44].

Fluoroquinolone resistance further constrains therapy [45]. Pre-pandemic ciprofloxacin resistance was higher in COVID-19 than non-COVID-19 units (46.2% vs. 38.9%). Iranian data show fluctuating *Klebsiella* sp. resistance and declines in *P. aeruginosa*, whereas other series reported increases peaking in 2020–2021 [46]. These trends were not observed in our setting, likely reflecting effective local stewardship measures and substantial reductions in ciprofloxacin use [47]. Persistently high rates in CAIC (~75% pre/in-pandemic) may be consistent with high-risk clones and biofilm-associated persistence [48,49,50,51]. Given hospital-wide resistance >20%, ciprofloxacin is unsuitable for empiric therapy of nosocomial infections.

Colistin, a last-line option, averaged 10.1% resistance in COVID-19 units vs. 2.4% in non-COVID-19 units pre-pandemic. Both rose during the pandemic (13.5% vs. 3.7%) and declined thereafter but remained higher in COVID-19 wards (8.4% vs. 2.6%). Our values mirror global averages (~3.1%) and the pandemic-era rise (~12.9%) [52,53]. Greater increases on repurposed wards likely reflect treatment of superinfections in critically ill patients and frequent isolation of highly resistant *Acinetobacter* sp. [54]. Carbapenemase producers often display higher colistin resistance; in our COVID-19 units, carbapenemase prevalence rose sharply during the pandemic [55]. Notably, CAIC averaged 18.3%, approaching the 20% threshold at which use of last-line agent becomes uncertain; infections with concurrent carbapenem and colistin resistance are exceptionally challenging given limited alternatives [56].

*Acinetobacter* sp. remains one of the most resistant nosocomial pathogens [57]. Increasing resistance to last-line agents like meropenem and colistin is a critical global challenge [58]. Ampicillin/sulbactam remains a cornerstone of combination regimens [59].

According to the latest ECDC–WHO surveillance (2023 data), *Acinetobacter* sp. continues to demonstrate the highest levels of antimicrobial resistance among all Gram-negative pathogens in Europe. Nearly half of all invasive isolates are resistant to carbapenems, with the highest rates reported in southern and eastern European countries. Although some western and northern regions have achieved partial declines since 2021, overall resistance remains above 40% across the EU/EEA [6].

Before the pandemic, mean ampicillin/sulbactam resistance was comparable between COVID-19 and non-COVID-19 units (51.3% vs. 52.2%). Resistance increased in both during the pandemic, slightly higher in non-COVID-19 units, and persisted post-pandemically. These values exceed previous Slovak data, and were generally higher in intensive care settings, reaching 72.1% in CAIC during the pandemic [60,61].

Significant increases occurred across multiple departments (DAIC, CAIC, 1st DS, 2nd DS, 1st DIM), indicating a hospital-wide trend. Similar pandemic-era patterns have been described internationally [62,63]. Rising ampicillin/sulbactam resistance is concerning as it diminishes its role in synergistic therapy [63].

The marked rise in ampicillin/sulbactam resistance from 2020 to 2022, without parallel consumption increases, suggests alternative drivers. Clonal analyses demonstrated *blaOXA-23* carriage in Brazilian carbapenem-resistant *Acinetobacter* sp. with high genetic similarity [63]. Similar mechanisms likely underlie concurrent increases in ampicillin/sulbactam and carbapenem resistance here. Pandemic-related ICU overload, weakened infection control, and suboptimal dosing (sulbactam ≥ 9 g/day rarely achieved) probably facilitated the spread of resistant clones independent of antibiotic pressure [63].

Overall, ampicillin/sulbactam resistance increase appears driven more by clonal expansion, environmental persistence, and pharmacodynamic inadequacy than by overuse.

Regarding colistin, global studies report rising resistance due to lipopolysaccharide modification, membrane permeability changes, efflux activity, and hetero-resistance [64]. Reports from Italy and Iran documented post-pandemic increases up to 44.2% [62,65]. In contrast, our data show persistently low colistin resistance despite increased use in COVID-19 units. The stability of susceptible clones lacking key resistance mutations (e.g., *pmrAB*, *lpxACD*) likely explains this observation, suggesting that local clonal dynamics, rather than global selective trends, shaped resistance patterns [66].

*P. aeruginosa* is a frequent nosocomial pathogen, accounting for ≈10–11% of infections and up to 23% in intensive care [67]. Its resistance mechanisms mainly involve porin modification and efflux pump overexpression [68]. According to the 2023 ECDC–WHO surveillance data, *P. aeruginosa* remains a critical therapeutic concern across Europe. Carbapenem resistance exceeds 40% in parts of southern and eastern Europe, while the EU/EEA mean remains above 20%. Although some northern and western countries reported slight post-2021 improvements, resistance to key agents—including carbapenems, fluoroquinolones, and aminoglycosides—persists at clinically significant levels, especially in intensive care environments [6].

In our study, resistance to cefepime declined across most departments, reaching statistical significance in both COVID-19 (CAIC, 2nd DS) and non-COVID-19 units (DAIC, 1st DIM). Baseline resistance exceeded 60% in CAIC and 40% in 1st DIM. Italian data reported lower baseline values (28%) with only a mild pandemic increase to 38%, contrasting with our findings [69]. In our hospital, pre-pandemic cefepime use was minimal, suggesting that direct selective pressure was unlikely. The persistent post-pandemic decline across all sites likely reflects clinical omission. Comparable results from Serbia demonstrated that cefepime withdrawal reduced resistance not only to cefepime but also to meropenem, piperacillin/tazobactam, and ceftazidime [70]. These effects are attributed to the attenuation of efflux-pump and porin-mediated mechanisms [71].

Meropenem resistance showed complex dynamics. Before the pandemic, resistance was low in non-COVID-19 wards (1st DIM, 1st DS) and the 4th DIM but markedly higher in the CAIC (73.6%) and DAIC (31.5%). Rates remained largely stable during the pandemic, contrasting reports of doubling in other ICUs [72]. This stability likely reflects effective stewardship that limited efflux-related carbapenem resistance [71,73].

Piperacillin/tazobactam resistance was initially highest in the CAIC (64%) and 2nd DS (38.9%) but declined during the pandemic to 36% and 30%, respectively, reaching statistical significance in the CAIC. This trend aligns with studies linking reductions to strict infection control despite increased antibiotic use [69,74]. Resistance stability, despite higher consumption, supports findings that extended-infusion dosing maintains efficacy and suppresses resistance [40].

Ciprofloxacin resistance decreased across several units, paralleling reduced use. However, it remained the only antibiotic with resistance consistently > 20% in all sites and years, reflecting extensive prior fluoroquinolone use and the intrinsic adaptability of *P. aeruginosa* [75].

Gentamicin and amikacin resistance declined across multiple departments, with the CAIC maintaining the highest and the 1st DS the lowest levels. International patterns vary, showing stability in Kuwait but ICU increases in Mexico [76,77]. Our decrease likely reflects reduced aminoglycoside use during the pandemic, consistent with Taipei data linking lower consumption to reduced resistance [40].

Analysis of resistance mechanisms showed that *Klebsiella* sp. exhibited declining ESBL prevalence and rising carbapenemase-mediated resistance. Non-COVID-19 units had more narrow-spectrum β-lactamases, whereas COVID-19 units demonstrated greater carbapenemase production, aminoglycoside resistance, and multidrug resistance. The ESBL decline parallels global reports and is linked to strengthened infection control, reduced antibiotic use, and travel restrictions [78]. Lower fluoroquinolone use likely contributed through the co-localisation of ESBL and fluoroquinolone resistance genes [79]. The rise in carbapenem resistance mirrors global trends of increasing CRE incidence during the pandemic, driven by intensive carbapenem use, prolonged ICU stays, and widespread immunosuppressive therapy [78,80,81].

The higher prevalence of narrow-spectrum β-lactamases in non-COVID-19 units is noteworthy. Literature suggests that enzymes such as TEM-1, SHV-1, and OXY-1, when combined with outer membrane protein mutations, can confer resistance to cefoperazone/sulbactam and piperacillin/tazobactam in otherwise carbapenem-susceptible *Enterobacteriaceae* [31]. Although Omp mutations were not evaluated in this study, the substantial increase in piperacillin/tazobactam and cefoperazone/sulbactam use during the pandemic—particularly in the DAIC—supports the hypothesis that these mechanisms underlie the observed rise in β-lactam resistance without a concurrent increase in carbapenem resistance.

For *Acinetobacter* sp., no major changes in carbapenem resistance were noted. For *P. aeruginosa*, we found significant decreasing trends in efflux + porin mechanisms, carbapenemase/MBL production, overall carbapenem resistance, and multidrug resistance within COVID-19 units.

The repurposing of hospitals significantly impacted antibiotic consumption. COVID-19 units showed markedly greater antibiotic consumption (e.g., meropenem use sharply increased during 2020–2021), indicating stronger selective pressure [82,83]. Trends in cephalosporin and fluoroquinolone use mirrored global patterns, with initial increases in some cephalosporins followed by stabilization or decline, and an overall reduction in fluoroquinolone use [84,85,86]. Higher baseline consumption in COVID-19 units, reflecting patient complexity and prescribing habits, was amplified during repurposing. Decrease in ciprofloxacin use showed a significant positive correlation with ESBL prevalence, while meropenem use correlated with carbapenemase resistance (ρ = 0.750; *p* = 0.052). Despite a marked decline in ceftriaxone use, no significant association with ESBL reduction was observed.

The comparison of clinically applicable antibiotics revealed substantial inter-unit disparities. Clinical applicability is increasingly limited by resistance, contributing to longer hospital stays, higher morbidity, mortality, and costs [87]. Inappropriate prescribing remains a major driver, with a significant share of inpatient antibiotic use failing to meet standards for indication, dosage, or duration [88]. The non-COVID-19 units had already implemented stricter ASP measures pre-pandemically, contributing to their more favourable resistance profiles.

Non-COVID-19 units (1st DIM, 1st DS, DAIC) retained a wider range of effective antibiotics, including piperacillin/tazobactam, cefoperazone/sulbactam, and meropenem against *Klebsiella* sp. and *P. aeruginosa*. For *Acinetobacter* sp., colistin remained the only effective option in both settings. COVID-19 units (4th DIM, 2nd DS, CAIC) were severely limited, with some wards having only one to four usable agents. High resistance rendered piperacillin/tazobactam, cefoperazone/sulbactam, meropenem, and aminoglycosides largely ineffective, while ceftriaxone and ciprofloxacin had lost all clinical value.

These discrepancies likely stem from differences in ASP enforcement, use of infection biomarkers, individualised PK/PD dosing, and infection-control standards [89].

Our study provides a unique contribution to AMR–COVID-19 research through its seven-year longitudinal scope (2018–2024) and granular, unit-level evaluation of resistance and antibiotic consumption. By directly comparing COVID-19 and non-COVID-19 units within a single hospital, it captures real-world stewardship and epidemiological dynamics often overlooked in multicenter or surveillance-based studies. The integration of consumption and mechanistic data allows clearer identification of selective pressures, offering valuable guidance for future stewardship and infection-control strategies.

This study has several limitations. A key limitation is the retrospective, single-center design, which constrains external validity and precludes causal inference from ecological associations between antibiotic consumption and resistance. Temporal changes unrelated to the pandemic such as evolving case-mix, shifts in diagnostic intensity, and variable device utilization, may confound observed trends, and we lacked patient-level covariates to adjust for these factors. We did not perform molecular typing; hence, inferences about clonal spread or specific mechanisms remain indirect. Antibiotic use was standardized as packages per 100 hospitalizations rather than DDD per 1000 patient-days, which may bias comparisons across wards with different lengths of stay and acuity, and dosing/PK–PD adherence (including extended infusions or sulbactam dose optimization) could not be verified. We analyzed clinical isolates without systematically distinguishing colonization from infection across all sites and time periods, and changes in screening or sampling practices during surge phases could introduce bias. Several subgroup analyses had limited sample size, reducing statistical power. Finally, we did not link resistance patterns to clinical outcomes (e.g., time to effective therapy, mortality), which limits conclusions about the patient-level impact of the observed epidemiology.

Nevertheless, our study provides valuable insight into local antimicrobial epidemiology and resistance dynamics. Future multicenter prospective studies with molecular typing are warranted to validate these results and to clarify transmission pathways. Linking resistance data with antibiotic consumption and stewardship interventions would further enhance clinical practice and optimize empiric therapy.

## 4. Materials and Methods

### 4.1. Study Design

This research, aimed at evaluating the prevalence and trends of resistance in the three most epidemiologically and clinically significant Gram-negative pathogens—*Klebsiella* sp., *Acinetobacter* sp., and *P. aeruginosa*—across selected departments of Louis Pasteur University Hospital (UNLP), was conducted as a retrospective observational study. The primary data source consisted of microbiological records from the Institute of Medical and Clinical Microbiology (IMCM), including antibiogram results and resistance profile analyses.

The analyzed data, spanning the period from 1 January 2018, to 31 December 2024, were divided into three time intervals:

- Pre-pandemic period (1 January 2018–31 December 2019);
- Pandemic period (1 January 2020–31 December 2022);
- Post-pandemic period (1 January 2023–31 December 2024).

Since data from the IMCM are evaluated on a calendar-year basis, they closely but not entirely reflect the pandemic timeframe. This division enabled monitoring of resistance dynamics in relation to prevailing epidemiological conditions, with an anticipated impact of the pandemic on antimicrobial resistance (AMR) trends.

### 4.2. Study Population

Two groups of hospital units were included in the analysis:

- COVID-19 units: These were repurposed during the pandemic to admit and manage patients with COVID-19. Units were selected based on sufficient numbers of hospitalized patients, bacterial isolates, and a diverse patient population. The following were included: the 4th Department of Internal Medicine (4th DIM), the 2nd Department of Surgery (2nd DS), and the Department of Anesthesiology and Intensive Care Medicine (DAIC);
- non-COVID-19 units: These departments, which had comparable patient numbers and case mix to the COVID-19 units, were not affected by the increased burden of COVID-19 hospitalizations. The following were included: the 1st Department of Internal Medicine (1st DIM), the 1st Department of Surgery (1st DS), and the Clinic of Anesthesiology and Intensive Care Medicine (CAIC).

Other departments were excluded primarily due to insufficient numbers of bacterial isolates. Patient counts in internal medicine and surgical units also encompassed individuals from their respective intensive care units.

The COVID-19 and non-COVID-19 units were located at separate hospital sites, which significantly limited patient transfers between them. This spatial separation minimized cross-unit patient mixing, thereby reducing the risk of direct microbial transmission. Consequently, the microbial environments of the respective sites were expected to exhibit distinct characteristics, shaped by differences in patient populations, antimicrobial exposure, and disinfection or sterilization practices. This factor plays a critical role in interpreting data on microbial ecology and the spectrum of nosocomial pathogens across hospital sites.

### 4.3. Methodology

The study was conducted as a retrospective analysis of epidemiological data, including evaluation of hospitalization numbers across departments using the Hospital Information System (HIS). Analyses were based on patient counts rather than hospital stays. Owing to HIS limitations, only so-called first admissions were assessed (patients with multiple admissions in a single year were counted once). Transfers between departments with subsequent returns were recorded as a single hospitalization. In collaboration with the hospital’s IT department, data were extracted under the technical constraints of the HIS, ensuring values were as close to reality as possible. The data were then systematically stratified by unit and year.

Microbiological data were obtained from the IMCM at UNLP. Analyses included total numbers of pathogens isolated from clinical samples, as well as resistance profiles. All clinical samples collected at the respective units were included, regardless of whether the isolated pathogen represented colonization or infection. This strategy was chosen to provide a comprehensive overview of the hospital microbiome.

Resistance to selected antibiotics was assessed using standard disk diffusion methods (Oxoid Ltd., Basingstoke, UK) and automated susceptibility testing using the VITEK 2 system (bioMérieux, Marcy-l’Étoile, France; software version 9.02). All procedures were carried out in accordance with the recommendations of the European Committee on Antimicrobial Susceptibility Testing (EUCAST, version 13.0, 2023). Resistance was reported as percentage values derived from antibiograms. Classification of pathogens as resistant or susceptible applied a 20% threshold, based on the average resistance value across the entire study period. Only resistance percentages were evaluated, not susceptibility. This approach enabled identification of clinically relevant resistance rates, evaluation of temporal dynamics, and comparison between COVID-19 and non-COVID-19 units.

The study focused on the following resistance mechanisms: production of narrow-spectrum β-lactamases, extended-spectrum β-lactamases (ESBL), AmpC, carbapenemases, aminoglycoside resistance, efflux pump activity, porin expression, and overall multidrug resistance. These mechanisms allowed for a comprehensive assessment of the type and dynamics of resistance during the study period.

Antibiotics included in resistance evaluation were as follows:

- *Acinetobacter* sp.: ampicillin/sublactam (SAM), piperacillin/tazobactam (TZP), ceftazidime (CAZ), cefepime (FEP), imipenem (IMP), meropenem (MEM), gentamicin (GEN), amikacin (AMI), ciprofloxacin (CIP), and colistin (COL);
- *P. aeruginosa*: TZP, CAZ, cefoperazone/sulbactam (SPZ), FEP, aztreonam (ATM), IMP, MEM, GEN, AMI, CIP, and COL;
- *Klebsiella* sp.: TZP, cefotaxime (CTX), CAZ, SPZ, FEP, ertapenem (ETP), MEM, GEN, AMI, CIP, and COL.

Antibiotic consumption data were obtained from the hospital pharmacy. Because consumption data were unavailable in the format of Defined Daily Doses (DDD), as recommended by the WHO, this methodology could not be applied. The limitation was due to the unavailability of precise milligram-level dispensing records for each antibiotic. Instead, the number of packages of each antibiotic consumed per 100 hospitalizations was chosen as a surrogate indicator, enabling relative comparisons across units. Identical drug package formats were used across all years, ensuring data comparability. This approach allowed monitoring of antibiotic consumption trends in the hospital setting despite the lack of standardized DDD data.

### 4.4. Statistical Analysis

Trends in hospitalizations and antibiotic resistance were analyzed using linear regression and Spearman’s rank correlation. Linear regression was applied to quantify linear trends (increase or decrease) in continuous variables over time. To assess monotonic trends, Spearman’s correlation was used, as it does not assume normal data distribution and is robust to outliers and small sample sizes. Statistical analyses were conducted using IBM SPSS Statistics (version 29.0; IBM Corp., Armonk, NY, USA).

Comparisons of hospitalization numbers between COVID-19 and non-COVID-19 units of the same type were performed using the paired parametric *t*-test, selected for its suitability in analyzing paired datasets from comparable units under different conditions. To verify robustness and account for the limited number of years analyzed, the non-parametric Wilcoxon signed-rank test was also applied.

For comparing average annual numbers of pathogen isolates across different time periods, the two-sample *t*-test with Welch’s correction was used, ensuring reliable results in the presence of unequal variances. Differences in isolate occurrence normalized per number of hospitalizations were evaluated using the non-parametric Mann–Whitney U test, due to potential non-normality in smaller sample sets.

Differences in the occurrence of selected nosocomial pathogens between COVID-19 and non-COVID-19 units were analyzed using Fisher’s exact test, appropriate for categorical data (pathogen present/absent) and small sample sizes.

All statistical methods were selected with respect to the characteristics of the analyzed data, sample size, and test assumptions. Since normality could not always be guaranteed due to limited sample sizes, a combination of parametric and non-parametric methods was employed to ensure robust results. Statistical significance was set at *p* < 0.05.

The results are presented in structured tables and figures illustrating observed trends and comparisons. The study was approved by the UNLP Ethics Committee (approval no. 2025/EK/04034).

## 5. Conclusions

This study demonstrates that the COVID-19 pandemic substantially influenced hospital activity, antibiotic consumption, and antimicrobial resistance, with distinct differences between COVID-19 and non-COVID-19 units. Hospitalizations in repurposed COVID-19 wards declined sharply during pandemic peaks, while intensive care units faced surges in admissions. Pathogen occurrence and resistance patterns were consistently higher in COVID-19 units, particularly for *Acinetobacter* sp., but also for *Klebsiella* sp. and *P. aeruginosa*.

Time-trend analyses revealed several significant decreases in pathogen occurrence and resistance, suggesting that long-term infection control measures and stewardship interventions achieved measurable effects. However, pandemic-related repurposing, increased broad-spectrum antibiotic use, and weakened infection-control practices contributed to surges in resistance, especially against carbapenems and β-lactam/β-lactamase inhibitor combinations. The most concerning finding was the limited clinical applicability of key antibiotics in COVID-19 units, where treatment options for multidrug-resistant pathogens were severely restricted.

In contrast, non-COVID-19 units maintained a broader spectrum of effective antibiotics, reflecting stricter antimicrobial stewardship and a more stable patient population. These results emphasize the critical role of stewardship, optimized prescribing, and rigorous infection prevention in preserving antibiotic effectiveness. Strengthening these measures is essential to mitigate the long-term consequences of any pandemic on antimicrobial resistance and to sustain the clinical utility of last-line agents in high-risk hospital settings. Moreover, the findings of this study provide a framework for future preparedness, offering practical guidance on antimicrobial management, infection control, and surveillance strategies that should be prioritized in the event of future pandemics.

## Figures and Tables

**Table 1:** Hospitalization trends in COVID-19 and non-COVID-19 units (2018–2024).

Unit	Department *	Regression Slope (b)	*p*-Value	Spearman’s ρ	*p*-Value
**COVID-19**	4th DIM	−99.7	**0.019**	−0.90	**0.037**
	2nd DS	−147.5	**0.028**	−0.90	**0.037**
	CAIC	5.8	0.761	−0.10	0.872
**non-COVID-19**	1st DIM	136.0	0.182	0.80	0.104
	1st DS	12.8	0.916	0.20	0.747
	DAIC	−5.4	0.129	−0.70	0.188

* Abbreviations of hospital departments: 4th DIM—4th Department of Internal Medicine; 2nd DS—2nd Department of Surgery; CAIC—Clinic of Anesthesiology and Intensive Care; 1st DIM—1st Department of Internal Medicine; 1st DS—1st Department of Surgery; DAIC—Department of Anesthesiology and Intensive Care.

**Table 2:** Significant trends in the occurrence of monitored isolates across individual departments.

Pathogen	Department *	Trend	Spearman’s ρ	*p*-Value
*Acinetobacter* sp.	4th DIM	↓	−0.857	**0.0137**
*Acinetobacter* sp.	CAIC	↓	−0.857	**0.0137**
*Klebsiella* sp.	1st DIM	↓	−0.893	**0.0068**
*Klebsiella* sp.	4th DIM	↓	−0.821	**0.0234**
*P. aeruginosa*	2nd DS	↓	−0.893	**0.0068**

* Abbreviations of hospital departments: 4th DIM—4th Department of Internal Medicine; 2nd DS—2nd Department of Surgery; CAIC—Clinic of Anesthesiology and Intensive Care; 1st DIM—1st Department of Internal Medicine.

**Table 3:** Comparison of pathogen occurrence in COVID-19 and non-COVID-19 units during the pandemic (2020–2022).

Pathogen	Year	COVID-19 Units (%)	Non-COVID-19 Units (%)	Odds Ratio (OR)	*p*-Value
*Acinetobacter* sp.	2020	8.25	1.78	4.97	**<0.001**
	2021	16.41	2.28	8.41	**<0.001**
	2022	9.93	2.24	4.81	**<0.001**
*Klebsiella* sp.	2020	13.17	7.38	1.90	**<0.001**
	2021	13.10	6.58	2.14	**<0.001**
	2022	17.48	5.92	3.37	**<0.001**
*P. aeruginosa*	2020	13.21	5.07	2.85	**<0.001**
	2021	9.52	3.54	2.86	**<0.001**
	2022	11.48	4.16	2.99	**<0.001**

**Table 4:** Significant trends of *Klebsiella* sp. resistance during the study period (2018–2024).

Unit	Department *	Antibiotic	Direction of Trend	Spearman’s ρ	*p*-Value
**COVID-19**	4th DIM	GEN	↓	–0.821	**0.023**
	2nd DS	ETP	↑	0.900	**0.037**
		SPZ	↑	0.900	**0.037**
	CAIC	GEN	↓	–0.857	**0.014**
**non-COVID-19**	1st DIM	GEN	↓	–0.857	**0.014**
	1st DS	AMI	↑	0.900	**0.037**
	DAIC	TZP	↑	0.857	**0.014**
		ETP	↑	0.893	**0.007**
		SPZ	↑	0.900	**0.037**
		MEM	↑	0.900	**0.037**
		COL	↑	0.900	**0.037**
		GEN	↓	–0.900	**0.037**

* Abbreviations of hospital departments: 4th DIM—4th Department of Internal Medicine; 2nd DS—2nd Department of Surgery; CAIC—Clinic of Anesthesiology and Intensive Care; 1st DIM—1st Department of Internal Medicine; 1st DS—1st Department of Surgery; DAIC—Department of Anesthesiology and Intensive Care.

**Table 5:** Significant trends of *Acinetobacter* sp. resistance during the study period (2018–2024).

Unit	Department *	Antibiotic	Direction of Trend	Spearman’s ρ	*p*-Value
**COVID-19**	2nd DS	SAM	↑	0.900	**0.037**
	CAIC	CAZ	↓	−0.786	**0.036**
		GEN	↓	−0.821	**0.023**
**non-COVID-19**	1st DIM	SAM	↑	0.943	**0.005**
	1st DS	SAM	↑	1.000	**0.0001**
	DAIC	SAM	↑	0.943	**0.005**
		GEN	↑	0.829	**0.042**
		AMI	↑	0.829	**0.042**

* Abbreviations of hospital departments: 2nd DS—2nd Department of Surgery; CAIC—Clinic of Anesthesiology and Intensive Care; 1st DIM—1st Department of Internal Medicine; 1st DS—1st Department of Surgery; DAIC—Department of Anesthesiology and Intensive Care.

**Table 6:** Significant trends of *P. aeruginosa* resistance during the study period (2018–2024).

Unit	Department *	Antibiotic	Direction of Trend	Spearman’s ρ	*p*-Value
**COVID-19**	2nd DS	FEP	↓	−0.857	**0.014**
		GEN	↓	−0.821	**0.023**
		CIP	↓	−0.786	**0.036**
	CAIC	TZP	↓	−0.964	**0.001**
		FEP	↓	−0.857	**0.014**
		GEN	↓	−0.857	**0.014**
**non-COVID-19**	1st DIM	CIP	↓	−0.821	**0.023**
		FEP	↓	−0.786	**0.036**
		GEN	↓	−0.786	**0.036**
	DAIC	FEP	↓	−0.901	**0.006**
		IMP	↓	−0.964	**0.001**
		GEN	↓	−0.786	**0.036**
		CIP	↓	−0.857	**0.014**

* Abbreviations of hospital departments: 1st DIM—1st Department of Internal Medicine; 2nd DS—2nd Department of Surgery; CAIC—Clinic of Anesthesiology and Intensive Care; DAIC—Department of Anesthesiology and Intensive Care.

**Table 7:** Inter-unit differences in resistance mechanisms (COVID-19 vs. non-COVID-19 units, 2018–2022).

Pathogen	Resistance Mechanisms	Direction of Difference	*p*-Value
***Klebsiella* sp.**	Narrow-spectrum β-lactamase	Higher in non-COVID-19 units	**0.007**
	Carbapenemase/MBL production	Higher in COVID-19 units	**≤0.007**
	Aminoglycoside resistance	Higher in COVID-19 units	**≤0.007**
	Multidrug resistance	Higher in COVID-19 units	**≤0.007**
***Acinetobacter* sp.**	Carbapenem resistance	Higher in COVID-19 units	**0.008**
	Multidrug resistance	Higher in COVID-19 units	**0.008**
***P. aeruginosa***	Efflux + porin (MexA,B-OprM + D-)	Higher in COVID-19 units	**0.032**
	Carbapenemase/MBL production	Higher in COVID-19 units	**0.008**
	Carbapenem resistance	Higher in COVID-19 units	**0.016**
	Multidrug resistance	Higher in COVID-19 units	**0.032**

**Table 8:** Correlation of antibiotic consumption and resistance mechanisms in *Klebsiella* sp.

Unit	Antibiotic *	Consumption	Mechanism	Resistance	*p*-Value
**COVID-19**	CTX	↓ 44.6%	ESBL	↓ 67.5%	0.667
	CIP	↓ 82.4%	ESBL	↓ 67.5%	**0.037**
	MEM	↑ 77.4%	Carbapenemase/MBL	↑ 84.3%	**0.052**
**non-COVID-19**	CTX	↓ 15.2%	ESBL	↓ 73.8%	ns
	CIP	↓ 85.9%	ESBL	↓ 73.8%	**0.016**

* CTX—cefotaxime, CIP—ciprofloxacin, MEM—meropenem, ESBL—extended-spectrum β-lactamase, MBL—metallo-β-lactamases, ns—not significant.

## Data Availability

The raw data supporting the conclusions of this article will be made available by the authors on request.

## Abbreviations

The following abbreviations are used in this manuscript:

1st DIM	1st Department of Internal Medicine
1st DS	1st Department of Surgery
2nd DS	2nd Department of Surgery
4th DIM	4th Department of Internal Medicine
AMI	amikacin
AMR	antimicrobial resistance
ASP	antimicrobial stewardship program
ATB	antibiotic
ATM	aztreonam
CAIC	Clinic of Anesthesiology and Intensive Care Medicine
CAZ	ceftazidime
CDC	Center for disease control and prevention
CIP	ciprofloxacin
COL	colistin
COVID-19	Coronavirus Disease 2019
CRE	carbapenem-resistant *Enterobacterales*
CR-AB	carbapenem-resistant *Acinetobacter baumannii*
CR-PA	carbapenem-resistant *Pseudomonas aeruginosa*
CTX	cefotaxime
DAIC	Department of Anesthesiology and Intensive Care Medicine
DDD	Defined Daily Doses
ESBL	extended spectrum β-lactamase
ETP	ertapenem
EU	European Uninon
EUCAST	European Committee on Antimicrobial Susceptibility Testing
FEP	cefepime
GEN	gentamicin
HIS	Hospital Information System
ICU	intensive care unit
IMCM	Institute of Medical and Clinical Microbiology
IMP	imipenem
MBL	metalo-β-lactamase
MDR	multi-drug resistant
MEM	meropenem
MIC	minimal inhibitory concentration
MRSA	methicillin-resistant *Staphylococcus aureus*
SAM	ampicillin/sulbactam
SPZ	cefoperazone
TZP	piperacillin/tazobactam
UNLP	Louis Pasteur University Hospital
UTI	urinary tract infection
VRE	vancomycin-resistant *Enterococcus*

## References

[1] Langford B.J., So M., Raybardhan S., Leung V., Westwood D., MacFadden D.R., Soucy J.-P.R., Daneman N. (2020). Bacterial co-infection and secondary infection in patients with COVID-19: A living rapid review and meta-analysis. Clin. Microbiol. Infect..

[2] Vermeulen H., Hens N., Catteau L., Catry B., Coenen S. (2023). Impact of the COVID-19 pandemic on community antibiotic consumption in the EU/European Economic Area: A changepoint analysis. J. Antimicrob. Chemother..

[3] Impact on Antimicrobial Resistance, Special Report 2022. https://www.cdc.gov/antimicrobial-resistance/media/pdfs/covid19-impact-report-508.pdf.

[4] Langford B.J., So M., Simeonova M., Leung V., Lo J., Kan T., Raybardhan S., Sapin M.E., Mponponsuo K., Farrell A. (2023). Antimicrobial resistance in patients with COVID-19: A systematic review and meta-analysis. Lancet Microbe.

[5] Calderon M., Gysin G., Gujjar A., McMaster A., King L., Comandé D., Hunter E., Payne B. (2023). Bacterial co-infection and antibiotic stewardship in patients with COVID-19: A systematic review and meta-analysis. BMC Infect. Dis..

[6] Antimicrobial Resistance in the EU/EEA (EARS-Net)—Annual Epidemiological Report 2023. https://www.ecdc.europa.eu/en/publications-data/antimicrobial-resistance-eueea-ears-net-annual-epidemiological-report-2023.

[7] Buehrle D.J., Decker B.K., Wagener M.M., Adalja A., Singh N., McEllistrem M.C., Nguyen M.H., Clancy C.J. (2020). Antibiotic Consumption and Stewardship at a Hospital outside of an Early Coronavirus Disease 2019 Epicenter. Antimicrob. Agents Chemother..

[8] Nieuwlaat R., Mbuagbaw L., Mertz D., Burrows L.L., Bowdish D.M.E., Moja L., Wright G.D., Schünemann H.J. (2021). Coronavirus Disease 2019 and Antimicrobial Resistance: Parallel and Interacting Health Emergencies. Clin. Infect. Dis..

[9] Arsenault C., Gage A., Kim M.K., Kapoor N.R., Akweongo P., Amponsah F., Aryal A., Asai D., Awoonor-Williams J.K., Ayele W. (2022). COVID-19 and resilience of healthcare systems in ten countries. Nat. Med..

[10] Nguyen J.L., Benigno M., Malhotra D., Khan F., Angulo F.J., Hammond J., Swerdlow D.L., Reimbaeva M., Emir B., McLaughlin J.M. (2022). Pandemic-related declines in hospitalization for non-COVID-19-related illness in the United States from January through July 2020. PLoS ONE.

[11] Lugli G., Ottaviani M.M., Botta A., Ascione G., Bruschi A., Cagnazzo F., Zammarchi L., Romagnani P., Portaluri T. (2022). The Impact of the SARS-CoV-2 Pandemic on Healthcare Provision in Italy to non-COVID Patients: A Systematic Review. Mediterr. J. Hematol. Infect. Dis..

[12] Habbous S., Lambrinos A., Petersen S., Hellsten E. (2023). The effect of the COVID-19 pandemic on hospital admissions and outpatient visits in Ontario, Canada. Ann. Thorac. Med..

[13] Filip R., Gheorghita Puscaselu R., Anchidin-Norocel L., Dimian M., Savage W.K. (2022). Global Challenges to Public Health Care Systems during the COVID-19 Pandemic: A Review of Pandemic Measures and Problems. J. Pers. Med..

[14] Berger E., Winkelmann J., Eckhardt H., Nimptsch U., Panteli D., Reichebner C., Rombey T., Busse R. (2022). A country-level analysis comparing hospital capacity and utilisation during the first COVID-19 wave across Europe. Health Policy.

[15] Sevalie S., Youkee D., van Duinen A.J., Bailey E., Bangura T., Mangipudi S., Mansaray E., Odland M.L., Parmar D., Samura S. (2021). The impact of the COVID-19 pandemic on hospital utilisation in Sierra Leone. BMJ Glob. Health.

[16] Baker M.A., Sands K.E., Huang S.S., Kleinman K., Septimus E.J., Varma N., Blanchard J., Poland R.E., Coady M.H., Yokoe D.S. (2022). The Impact of Coronavirus Disease 2019 (COVID-19) on Healthcare-Associated Infections. Clin. Infect. Dis..

[17] Karruli A., Boccia F., Gagliardi M., Patauner F., Ursi M.P., Sommese P., De Rosa R., Murino P., Ruocco G., Corcione A. (2021). Multidrug-Resistant Infections and Outcome of Critically Ill Patients with Coronavirus Disease 2019: A Single Center Experience. Microb. Drug Resist..

[18] Martin-Loeches I., Restrepo M.I. (2024). COVID-19 vs. non-COVID-19 related nosocomial pneumonias: Any differences in etiology, prevalence, and mortality?. Curr. Opin. Crit. Care.

[19] Bentivegna E., Luciani M., Arcari L., Santino I., Simmaco M., Martelletti P. (2021). Reduction of Multidrug-Resistant (MDR) Bacterial Infections during the COVID-19 Pandemic: A Retrospective Study. Int. J. Environ. Res. Public Health.

[20] Golli A.L., Popa S.G., Ghenea A.E., Turcu F.L. (2025). The Impact of the COVID-19 Pandemic on the Antibiotic Resistance of Gram-Negative Pathogens Causing Bloodstream Infections in an Intensive Care Unit. Biomedicines.

[21] Sulayyim H.J.A., Ismail R., Hamid A.A., Ghafar N.A. (2022). Antibiotic Resistance during COVID-19: A Systematic Review. Int. J. Environ. Res. Public Health.

[22] Altamimi I., Binkhamis K., Alhumimidi A., Alabdulkarim I.M., Almugren A., Alhemsi H., Altamimi A., Almazyed A., Elbih S., Alghunaim R. (2024). Decline in ESBL Production and Carbapenem Resistance in Urinary Tract Infections among Key Bacterial Species during the COVID-19 Pandemic. Antibiotics.

[23] Lee J., Pai H., Kim Y.K., Kim N.H., Eun B.W., Kang H.J., Park K.H., Choi E.H., Shin H.Y., Kim E.C. (2007). Control of extended-spectrum beta-lactamase-producing *Escherichia coli* and *Klebsiella pneumoniae* in a children’s hospital by changing antimicrobial agent usage policy. J. Antimicrob. Chemother..

[24] Struelens M.J. (1998). The epidemiology of antimicrobial resistance in hospital acquired infections: Problems and possible solutions. BMJ.

[25] Kolář M., Štrbová P. (2015). Vývoj rezistence invazivních bakterií v souvislosti se spotřebou antibiotic. Prakt. Lékáren..

[26] Luo C., Chen Q. (2025). Trends in CRKP Prevalence and Risk Factors for CRKP Hospital-Acquired Infections in Pediatric Patients Pre-, During-, and Post-COVID-19 Pandemic. Microb. Drug Resist..

[27] Kolbe-Busch S., Djouela Djoulako P.D., Stingu C.S. (2025). Trends in Healthcare-Acquired Infections Due to Multidrug-Resistant Organisms at a German University Medical Center Before and During the COVID-19 Pandemic. Microorganisms.

[28] Yang X., Liu X., Li W., Shi L., Zeng Y., Xia H., Huang Q., Li J., Li X., Hu B. (2023). Epidemiological Characteristics and Antimicrobial Resistance Changes of Carbapenem-Resistant *Klebsiella pneumoniae* and *Acinetobacter baumannii* under the COVID-19 Outbreak: An Interrupted Time Series Analysis in a Large Teaching Hospital. Antibiotics.

[29] Önal U., Tüzemen Ü., Kazak E., Gençol N., Souleiman E., İmer H., Heper Y., Yılmaz E., Özakın C., Ener B. (2023). Effects of COVID-19 pandemic on healthcare-associated infections, antibiotic resistance and consumption rates in intensive care units. Infez. Med..

[30] O’Riordan F., Shiely F., Byrne S., O’Brien D., Ronayne A., Fleming A. (2022). Antimicrobial use and antimicrobial resistance in *Enterobacterales* and *Enterococcus faecium*: A time series analysis. J. Hosp. Infect..

[31] Yang F., Zhao Q., Wang L., Wu J., Jiang L., Sheng L., Zhang L., Xue Z., Yi M. (2022). Diminished Susceptibility to Cefoperazone/Sulbactam and Piperacillin/Tazobactam in *Enterobacteriaceae* Due to Narrow-Spectrum β-Lactamases as Well as Omp Mutation. Pol. J. Microbiol..

[32] Sharma A., Thakur A., Thakur N., Kumar V., Chauhan A., Bhardwaj N. (2023). Changing Trend in the Antibiotic Resistance Pattern of *Klebsiella pneumoniae* Isolated from Endotracheal Aspirate Samples of ICU Patients of a Tertiary Care Hospital in North India. Cureus.

[33] Rahimzadeh G., Rezai S., Abbasi G., Soleimanpour S., Valadan R., Vahedi L., Sheidaei S., Movahedi F.S., Rezai R., Rezai M.S. (2024). High prevalence of antimicrobial resistance genes in multidrug-resistant-ESBLs-producing *Klebsiella pneumoniae* post-COVID-19 pandemic. Iran. J. Microbiol..

[34] Chiang T.T., Chiang M.H., Tang H.J., Shi Z.Y., Ho M.W., Chou C.H., Lin S.Y., Lu P.L., Wu T.S., Shie S.S. (2024). study on clinical outcomes and poor prognostic factors in patients with *Klebsiella pneumoniae* bacteremia receiving cefoperazone/sulbactam treatment. Eur. J. Clin. Microbiol. Infect. Dis..

[35] Cireșă A., Tălăpan D., Vasile C.C., Popescu C., Popescu G.A. (2024). Evolution of Antimicrobial Resistance in *Klebsiella pneumoniae* over 3 Years (2019-2021) in a Tertiary Hospital in Bucharest, Romania. Antibiotics.

[36] Catalano A., Iacopetta D., Ceramella J., Pellegrino M., Giuzio F., Marra M., Rosano C., Saturnino C., Sinicropi M.S., Aquaro S. (2023). Antibiotic-Resistant ESKAPE Pathogens and COVID-19: The Pandemic beyond the Pandemic. Viruses.

[37] Nasiri G., Peymani A., Farivar T.N., Hosseini P. (2018). Molecular epidemiology of aminoglycoside resistance in clinical isolates of *Klebsiella pneumoniae* collected from Qazvin and Tehran provinces, Iran. Infect. Genet. Evol..

[38] Abu Lubad M.A., Abu-Helalah M.A., Al-Hajaia T.S., Al-Hutaibat K.A., Aqel A.A., Alzoubi H. (2023). COVID-19 pandemic impact on antibiotics sensitivity of *Escherichia coli* and *Klebsiella pneumoniae* from urine specimens: A retrospective study. J. Infect. Dev. Ctries..

[39] Hara G.L., Antik A., Aguirre S., Giuliano C., García D., Ochiuzzi M.E., Kanenguiser P., Prieto N., Fernández A., Neumann G. (2024). The effect of the COVID-19 pandemic on the incidence and resistance of Gram-negative bacilli and antimicrobial consumption in the intensive care units of a referral hospital in Buenos Aires. Int. J. Antimicrob. Agents.

[40] Huang H.W., Liu H.Y., Chuang H.C., Chen B.L., Wang E.Y., Tsao L.H., Ai M.Y., Lee Y.J. (2023). Correlation between antibiotic consumption and resistance of *Pseudomonas aeruginosa* in a teaching hospital implementing an antimicrobial stewardship program: A longitudinal observational study. J. Microbiol. Immunol. Infect..

[41] Kacířová I., Grundmann M. (2015). Terapeutické monitorování amikacinu a gentamicinu v rutinní klinické praxi [Therapeutic monitoring of amikacin and gentamicin in routine clinical practice]. Vnitr. Lek..

[42] Kadri S.S., Adjemian J., Lai Y.L., Spaulding A.B., Ricotta E., Prevots D.R., Palmore T.N., Rhee C., Klompas M., Dekker J.P. (2018). Difficult-to-Treat Resistance in Gram-negative Bacteremia at 173 US Hospitals: Retrospective Cohort Analysis of Prevalence, Predictors, and Outcome of Resistance to All First-line Agents. Clin. Infect. Dis..

[43] Swedan S., Alabdallah E.A., Ababneh Q. (2023). Resistance to aminoglycoside and quinolone drugs among *Klebsiella pneumoniae* clinical isolates from northern Jordan. Heliyon.

[44] Ramirez M.S., Tolmasky M.E. (2017). Amikacin: Uses, Resistance, and Prospects for Inhibition. Molecules.

[45] Geetha P.V., Aishwarya K.V.L., Mariappan S., Sekar U. (2020). Fluoroquinolone Resistance in Clinical Isolates of *Klebsiella pneumoniae*. J. Lab. Physicians.

[46] Nabavi A.S., Abolghasemi S., Mardani M., Tfti M.F., Talebian N., Ghasemi R. (2025). Evaluating the Antibiotic Resistance Pattern Before and After the COVID-19 Pandemic Among Cancer Patients. Arch. Clin. Infect. Dis..

[47] Alshehri S.M., Abdullah N.S., Algarni A., AlZomia A.S., Assiry M.M. (2024). Resistance Pattern of *Klebsiella pneumoniae* in Aseer Region, Saudi Arabia: A Ten-Year Hospital-Based Study. Medicina.

[48] Paterson D.L., Mulazimoglu L., Casellas J.M., Ko W.C., Goossens H., Von Gottberg A., Mohapatra S., Trenholme G.M., Klugman K.P., McCormack J.G. (2000). Epidemiology of ciprofloxacin resistance and its relationship to extended-spectrum beta-lactamase production in *Klebsiella pneumoniae* isolates causing bacteremia. Clin. Infect. Dis..

[49] Karampatakis T., Tsergouli K., Behzadi P. (2023). Carbapenem-Resistant *Klebsiella pneumoniae*: Virulence Factors, Molecular Epidemiology and Latest Updates in Treatment Options. Antibiotics.

[50] Ostria-Hernandez M.L., Juárez-de la Rosa K.C., Arzate-Barbosa P., Lara-Hernández A., Sakai F., Ibarra J.A., Castro-Escarpulli G., Vidal J.E. (2018). Nosocomial, Multidrug-Resistant *Klebsiella pneumoniae* Strains Isolated from Mexico City Produce Robust Biofilms on Abiotic Surfaces but Not on Human Lung Cells. Microb. Drug Resist..

[51] Zahornacký O., Porubčin Š., Rovňáková A., Jarčuška P. (2022). Gram-Negative Rods on Inanimate Surfaces of Selected Hospital Facilities and Their Nosocomial Significance. Int. J. Environ. Res. Public Health.

[52] Antoniadou A., Kontopidou F., Poulakou G., Koratzanis E., Galani I., Papadomichelakis E., Kopterides P., Souli M., Armaganidis A., Giamarellou H. (2007). Colistin-resistant isolates of *Klebsiella pneumoniae* emerging in intensive care unit patients: First report of a multiclonal cluster. J. Antimicrob. Chemother..

[53] Uzairue L.I., Rabaan A.A., Adewumi F.A., Okolie O.J., Folorunso J.B., Bakhrebah M.A., Garout M., Alfouzan W.A., Halwani M.A., Alamri A.A. (2022). Global Prevalence of Colistin Resistance in *Klebsiella pneumoniae* from Bloodstream Infection: A Systematic Review and Meta-Analysis. Pathogens.

[54] Rangel K., Chagas T.P.G., De-Simone S.G. (2021). *Acinetobacter baumannii* Infections in Times of COVID-19 Pandemic. Pathogens.

[55] El-Mahallawy H.A., El Swify M., Abdul Hak A., Zafer M.M. (2022). Increasing trends of colistin resistance in patients at high-risk of carbapenem-resistant *Enterobacteriaceae*. Ann. Med..

[56] Popivanov G., Markovska R., Gergova I., Konaktchieva M., Cirocchi R., Kjossev K., Mutafchiyski V. (2024). An Intra-Hospital Spread of Colistin-Resistant *Klebsiella pneumoniae* Isolates—Epidemiological, Clinical, and Genetic Analysis. Medicina.

[57] Tacconelli E., Carrara E., Savoldi A., Harbarth S., Mendelson M., Monnet D.L., Pulcini C., Kahlmeter G., Kluytmans J., Carmeli Y. (2018). Discovery, research, and development of new antibiotics: The WHO priority list of antibiotic-resistant bacteria and tuberculosis. Lancet Infect. Dis..

[58] Islam M.M., Jung D.E., Shin W.S., Oh M.H. (2024). Colistin Resistance Mechanism and Management Strategies of Colistin-Resistant *Acinetobacter baumannii* Infections. Pathogens.

[59] Betrosian A.P., Frantzeskaki F., Xanthaki A., Georgiadis G. (2007). High-dose ampicillin-sulbactam as an alternative treatment of late-onset VAP from multidrug-resistant *Acinetobacter baumannii*. Scand. J. Infect. Dis..

[60] Jalali Y., Liptáková A., Jalali M., Payer J. (2023). Moving toward Extensively Drug-Resistant: Four-Year Antimicrobial Resistance Trends of *Acinetobacter baumannii* from the Largest Department of Internal Medicine in Slovakia. Antibiotics.

[61] Rhomberg P.R., Fritsche T.R., Sader H.S., Jones R.N. (2006). Antimicrobial susceptibility pattern comparisons among intensive care unit and general ward Gram-negative isolates from the Meropenem Yearly Susceptibility Test Information Collection Program (USA). Diagn. Microbiol. Infect. Dis..

[62] Ghamari M., Jabalameli F., Afhami S., Halimi S., Emaneini M., Beigverdi R. (2025). *Acinetobacter baumannii* infection in critically ill patients with COVID-19 from Tehran, Iran: The prevalence, antimicrobial resistance patterns and molecular characteristics of isolates. Front. Cell Infect. Microbiol..

[63] Chaiben V., Yamada C.H., Telles J.P., de Andrade A.P., Arend L.N.V.S., Ribeiro V.S.T., Dantas L.R., Suss P.H., Tuon F.F. (2022). A carbapenem-resistant *Acinetobacter baumannii* outbreak associated with a polymyxin shortage during the COVID pandemic: An in vitro and biofilm analysis of synergy between meropenem, gentamicin and sulbactam. J. Antimicrob. Chemother..

[64] Kamoshida G., Yamada N., Yamaguchi D., Yahiro K., Morita Y. (2025). Colistin Resistance in *Acinetobacter baumannii*: Basic and Clinical Insights. Biol. Pharm. Bull..

[65] Petazzoni G., Bellinzona G., Merla C., Corbella M., Monzillo V., Samuelsen Ø., Corander J., Sassera D., Gaiarsa S., Cambieri P. (2023). The COVID-19 Pandemic Sparked Off a Large-Scale Outbreak of Carbapenem-Resistant *Acinetobacter baumannii* from the Endemic Strains at an Italian Hospital. Microbiol. Spectr..

[66] Camargo C.H., Yamada A.Y., Nagamori F.O., de Souza A.R., Tiba-Casas M.R., de Moraes França F.A., Porto M.H.T.N., de Lima Garzon M.L., Higgins P., Madalosso G. (2022). Clonal spread of ArmA- and OXA-23-coproducing *Acinetobacter baumannii* International Clone 2 in Brazil during the first wave of the COVID-19 pandemic. J. Med. Microbiol..

[67] Vincent J.L., Sakr Y., Singer M., Martin-Loeches I., Machado F.R., Marshall J.C., Finfer S., Pelosi P., Brazzi L., Aditianingsih D. (2020). Prevalence and Outcomes of Infection Among Patients in Intensive Care Units in 2017. JAMA.

[68] Yang Y., Li X., Sun L., Wang X.-K., Zhang Y.-W., Pang J., Li G.-Q., Hu X.-X., Nie T.-Y., Yang X.-Y. (2025). High level non-carbapenemase carbapenem resistance by overlaying mutations of mexR, oprD, and ftsI in *Pseudomonas aeruginosa*. Microbiol. Spectr..

[69] Serretiello E., Manente R., Dell’Annunziata F., Folliero V., Iervolino D., Casolaro V., Perrella A., Santoro E., Galdiero M., Capunzo M. (2023). Antimicrobial Resistance in *Pseudomonas aeruginosa* before and during the COVID-19 Pandemic. Microorganisms.

[70] Djordjevic Z.M., Folic M.M., Jankovic S.M. (2018). Correlation between cefepime utilisation and *Pseudomonas aeruginosa* resistance rates to β-lactams and carbapenems in patients with healthcare-associated infections. J. Glob. Antimicrob. Resist..

[71] Hocquet D., Nordmann P., El Garch F., Cabanne L., Plésiat P. (2006). Involvement of the MexXY-OprM efflux system in emergence of cefepime resistance in clinical strains of *Pseudomonas aeruginosa*. Antimicrob. Agents Chemother..

[72] Despotovic A., Milosevic B., Cirkovic A., Vujovic A., Cucanic K., Cucanic T., Stevanovic G. (2021). The Impact of COVID-19 on the Profile of Hospital-Acquired Infections in Adult Intensive Care Units. Antibiotics.

[73] Butscheid Y., Frey P.M., Pfister M., Pagani L., Kouyos R.D., Scheier T.C., Staiger W.I., Mancini S., Brugger S.D. (2025). Decline of antimicrobial resistance in *Pseudomonas aeruginosa* bacteraemia following the COVID-19 pandemic: A longitudinal observational study. J. Antimicrob. Chemother..

[74] Xia J., Lu L., Zhao K.L., Zeng Q.L. (2023). Resistance Transition of *Pseudomonas aeruginosa* in SARS-CoV-2-Uninfected Hospitalized Patients in the Pandemic. Infect. Drug Resist..

[75] Su H.C., Ramkissoon K., Doolittle J., Clark M., Khatun J., Secrest A., Wolfgang M.C., Giddings M.C. (2010). The development of ciprofloxacin resistance in *Pseudomonas aeruginosa* involves multiple response stages and multiple proteins. Antimicrob. Agents Chemother..

[76] Alali W.Q., Abdo N.M., AlFouzan W., Dhar R. (2022). Antimicrobial resistance pattern in clinical *Escherichia coli* and *Pseudomonas aeruginosa* isolates obtained from a secondary-care hospital prior to and during the COVID-19 pandemic in Kuwait. Germs.

[77] López-Jácome L.E., Fernández-Rodríguez D., Franco-Cendejas R., Camacho-Ortiz A., Morfin-Otero M.D.R., Rodríguez-Noriega E., Ponce-de-León A., Ortiz-Brizuela E., Rojas-Larios F., Velázquez-Acosta M.D.C. (2022). Increment Antimicrobial Resistance During the COVID-19 Pandemic: Results from the Invifar Network. Microb. Drug Resist..

[78] Abubakar U., Al-Anazi M., Alanazi Z., Rodríguez-Baño J. (2023). Impact of COVID-19 pandemic on multidrug resistant gram positive and gram negative pathogens: A systematic review. J. Infect. Public Health.

[79] Talan D.A., Takhar S.S., Krishnadasan A., Abrahamian F.M., Mower W.R., Moran G.J., EMERGEncy ID Net Study Group (2016). Fluoroquinolone-Resistant and Extended-Spectrum β-Lactamase-Producing *Escherichia coli* Infections in Patients with Pyelonephritis, United States. Emerg. Infect. Dis..

[80] Duffy N., Li R., Czaja C.A., Johnston H., Janelle S.J., Jacob J.T., Smith G., Wilson L.E., Vaeth E., Lynfield R. (2023). Trends in Incidence of Carbapenem-Resistant Enterobacterales in 7 US Sites, 2016–2020. Open Forum Infect. Dis..

[81] Zhang J., Li Q., Liu J., Fan F., Shi Y., Yu X. (2025). Prevalence of hypervirulent *Klebsiella pneumoniae* strains in COVID-19 patients with bacterial co-infections. Front. Microbiol..

[82] da Silva C.F., Deutschendorf C., Nagel F.M., Dalmora C.H., Dos Santos R.P., Lisboa T.C. (2021). Impact of the pandemic on antimicrobial consumption patterns. Infect. Control Hosp. Epidemiol..

[83] Pandak N., Al Sidairi H., Al-Zakwani I., Al Balushi Z., Chhetri S., Ba’Omar M., Al Lawati S., Al-Abri S.S., Khamis F. (2023). The Outcome of Antibiotic Overuse before and during the COVID-19 Pandemic in a Tertiary Care Hospital in Oman. Antibiotics.

[84] Hussein R.R., Rabie A.S.I., Bin Shaman M., Shaaban A.H., Fahmy A.M., Sofy M.R., Lattyak E.A., Abuelhana A., Naguib I.A., Ashour A.M. (2022). Antibiotic consumption in hospitals during COVID-19 pandemic: A comparative study. J. Infect. Dev. Ctries..

[85] Serwacki P., Hareza D.A., Gajda M., Świątek-Kwapniewska W., Adamowska M., Serwacka K., Zawada G., Wałaszek M., Wójkowska-Mach J. (2025). Fluoroquinolone consumption and resistance after an Antibiotic Stewardship Team intervention—An interventional study in a single hospital in Southern Poland from 2018 to 2023. Am. J. Infect. Control.

[86] Vlad M.A., Iancu L.S., Dorneanu O.S., Duhaniuc A., Pavel-Tanasa M., Tuchilus C.G. (2025). Colistin Treatment Outcomes in Gram-Negative Bacterial Infections in the Northeast of Romania: A Decade of Change Through Pandemic Challenges. Antibiotics.

[87] Chinemerem Nwobodo D., Ugwu M.C., Oliseloke Anie C., Al-Ouqaili M.T.S., Chinedu Ikem J., Victor Chigozie U., Saki M. (2022). Antibiotic resistance: The challenges and some emerging strategies for tackling a global menace. J. Clin. Lab. Anal..

[88] Salam M.A., Al-Amin M.Y., Salam M.T., Pawar J.S., Akhter N., Rabaan A.A., Alqumber M.A.A. (2023). Antimicrobial Resistance: A Growing Serious Threat for Global Public Health. Healthcare.

[89] Schouten J., De Waele J., Lanckohr C., Koulenti D., Haddad N., Rizk N., Sjövall F., Kanj S.S., Alliance for the Prudent Use of Antibiotics (APUA) (2021). Antimicrobial stewardship in the ICU in COVID-19 times: The known unknowns. Int. J. Antimicrob. Agents.

